# Crystal structures and inhibition of *Trypanosoma brucei* hypoxanthine–guanine phosphoribosyltransferase

**DOI:** 10.1038/srep35894

**Published:** 2016-10-27

**Authors:** David Terán, Dana Hocková, Michal Česnek, Alena Zíková, Lieve Naesens, Dianne T. Keough, Luke W. Guddat

**Affiliations:** 1 The School of Chemistry and Molecular Biosciences, The University of Queensland, Brisbane, 4072 QLD, Australia; 2 The Institute of Organic Chemistry and Biochemistry of the Czech Academy of Sciences, Flemingovo nam. 2, CZ-166 10 Prague 6, Czech Republic; 3 Biology Centre CAS, Institute of Parasitology, Branišovská 31, České Budějovice, Czech Republic; 4 Rega Institute for Medical Research, KU Leuven–University of Leuven, Minderbroedersstraat 10, B-3000 Leuven, Belgium

## Abstract

Human African Trypanosomiasis (HAT) is a life-threatening infectious disease caused by the protozoan parasite, *Trypanosoma brucei* (*Tbr*). Due to the debilitating side effects of the current therapeutics and the emergence of resistance to these drugs, new medications for this disease need to be developed. One potential new drug target is 6-oxopurine phosphoribosyltransferase (PRT), an enzyme central to the purine salvage pathway and whose activity is critical for the production of the nucleotides (GMP and IMP) required for DNA/RNA synthesis within this protozoan parasite. Here, the first crystal structures of this enzyme have been determined, these in complex with GMP and IMP and with three acyclic nucleoside phosphonate (ANP) inhibitors. The K_i_ values for GMP and IMP are 30.5 μM and 77 μM, respectively. Two of the ANPs have K_i_ values considerably lower than for the nucleotides, 2.3 μM (with guanine as base) and 15.8 μM (with hypoxanthine as base). The crystal structures show that when two of the ANPs bind, they induce an unusual conformation change to the loop where the reaction product, pyrophosphate, is expected to bind. This and other structural differences between the *Tbr* and human enzymes suggest selective inhibitors for the *Tbr* enzyme can be designed.

*Trypanosoma brucei* is the protozoan parasite that is the causative agent of Human African Trypanosomiasis (HAT), also known as African sleeping sickness. HAT is a devastating and often fatal disease in humans and domestic animals that is endemic to 36 countries in sub-Saharan Africa^1^. The World Health Organization (WHO) estimates that 20,000 new cases of HAT occur each year with a further 65 million people at risk of infection^1^,^2^. HAT is transmitted to humans by the bite of an infected tsetse fly. Infected patients progress gradually to a coma and severe organ failure which ultimately is fatal^3^. Five therapeutic agents to treat this disease, *i.e.* pentamidine, eflornithine, nifurtimox, melarsoprol and suramin, are currently available. However, these are far from ideal since they have numerous severe side effects including hypoglycemia, hypotension, encephalopathic syndrome, peripheral neuropathy and hepatic toxicity^3^,^4^,^5^. In addition to these issues, treatment of HAT is becoming more problematic due to the emergence of resistance to these drugs. In the last decade the rate of failure of HAT treatment with melarsoprol is reported to be as high as 39%^4^,^6^,^7^. The recently developed drug, eflornithine, is also not considered to be an ideal therapy as it requires intravenous administration^3^,^4^,^5^,^6^. Thus, new targets within the *Trypanosoma brucei* parasite need to be identified to begin the development of new therapeutics for this neglected disease^8^.

One approach to progressing new drug discoveries is to target an enzymatic pathway whose activity is crucial to maintaining replication of the pathogen. Also, significant differences should exist in the structure and activity of the pathogen’s enzyme compared to that in the human host, so that such differences can be exploited to achieve selectivity. In humans there are two pathways for the synthesis of the purine nucleoside monophosphates required for DNA/RNA production. These are by *de novo* synthesis starting with simple precursor molecules and by salvage and recycling of the purine bases. However, in *T. brucei* there are no enzymes for *de novo* synthesis and this parasite relies solely on its salvage pathways^9^,^10^,^11^ to make its purine nucleoside monophosphates. The *T. brucei* genome project^12^ has identified many of the enzymes expected to play key roles in the recycling and salvage of purine bases and nucleosides. These include three genes for a 6-oxopurine phosphoribosyltransferase annotated as hypoxanthine-guanine phosphoribosyltransferase (HGPRT), two adenine phosphoribosyltransferases (APRT), two nucleoside hydrolases and an adenosine kinase as well as several enzymes responsible for nucleotide interconversion (*e.g.* an IMP dehydrogenase and a GMP synthetase)^5^,^13^,^14^,^15^. Evidently, there is clear redundancy in the salvage pathway enzymes, but since the parasite takes up the prevailing purine precursors (*i.e.* hypoxanthine, xanthine and inosine) from blood serum and cerebrospinal fluid, at least some of the enzymes responsible for the synthesis of GMP, AMP and IMP should be essential *in vivo*^16^. This is exemplified by a recent study by Li and coworkers that showed GMP synthase is essential for *T. brucei* virulence and viability *in vivo*^13^. Thus, blocking the activity of one or more of these appears to be a viable approach to preventing DNA/RNA production. HGPRT is central to this salvage pathway, as it is responsible for the synthesis of IMP and GMP from hypoxanthine and guanine, respectively (Fig. 1A). Therefore, investigation into the structure and inhibition of the protein products of all three HGPRT genes is essential information for drug development.

One class of compounds with potential as therapeutic drug leads for human protozoan infections are the acyclic nucleoside phosphonates (ANPs). These compounds (Fig. 1B) are analogs of the nucleoside monophosphate products of the HGPRT catalyzed reaction (Fig. 1A). An important feature of the ANPs is that they contain a stable carbon-phosphorus bond that links the phosphoryl moiety to the remainder of the molecule^17^,^18^. Thus, such compounds are not susceptible to hydrolysis within the cell of the host or pathogen. Several distinct ANP analogues have proven to be potent inhibitors of *Plasmodium falciparum* HGXPRT, *Plasmodium vivax* HGPRT, and *Mycobacterium tuberculosis* HGPRT, and prodrugs of these ANPs have antimalarial and antituberculosis activity^18^,^19^,^20^,^21^,^22^,^23^,^24^,^25^.

Here, to begin our understanding of the molecular basis for 6-oxopurine salvage in *Trypansoma brucei*, we have expressed, purified and characterized one of the isoforms of *Tbr*HGPRT and determined the inhibition constants of this enzyme for seven ANPs^19^. The crystal structures of this enzyme in complex with IMP and GMP and with three ANPs have been determined with the aim to use insights from this data to improve the design of the ANP inhibitors resulting in more potent activity.

## Results and Discussion

### Expression, purification and specific activity

The HGPRT, identified as Tb927.10.1400 in the *Tbr* genome data base (www.tritrypdb.org) with an N-terminal hexa-histidine tag attached to the polypeptide was expressed in *E. coli* cells and purified to homogeneity as assessed by SDS-PAGE (Supplementary Figure 1A). Approximately 10 mg of purified enzyme was obtained per litre of culture, with a specific activity of 70 μmol min^−1^ mg^−1^ when guanine is the substrate. This value is similar to that for this enzyme obtained in the absence of the tag, having a specific activity of 53 μmol min^−1^ mg^−1^, though this value was measured under slightly different assay conditions^26^. Thus, the hexa-His tag does not appear to affect the activity of this enzyme.

The kinetic constants of the naturally occurring base substrates for *Tbr* HGPRT are presented in Table 1, showing that guanine is the preferred substrate with the lowest K_m_ (2.3 μM) and the most rapid turnover value (k_cat_ = 23.8 s^−1^), and a k_cat_/K_m_ value that is 3.3-fold higher than that of hypoxanthine. Xanthine was also tested as a substrate but showed only very weak activity (Table 1 and Supplementary Figures 1D,E), confirming the annotation of this enzyme as an HGPRT. Comparing the activities of the substrates of this enzyme with that of *Trypanosoma cruzi* (*Tcr)* HGPRT shows that they have similar K_m_ values, all in the 2–10 μM range, and similar k_cat_ values in the range of 17–41 s^−1^ (Table 1)^27^. Thus, these two 6-oxopurine PRTs from two closely related biological species possess similar substrate profiles. By comparison, human HGPRT also has slight preference for guanine as the base substrate, though the k_cat_ values are 2–3 fold faster for the *Tbr* enzyme than the human enzyme. This difference suggests that there may be some structural variations between the human and parasite enzymes.

### Inhibition studies for *Tbr*HGPRT

**Figure 1:**
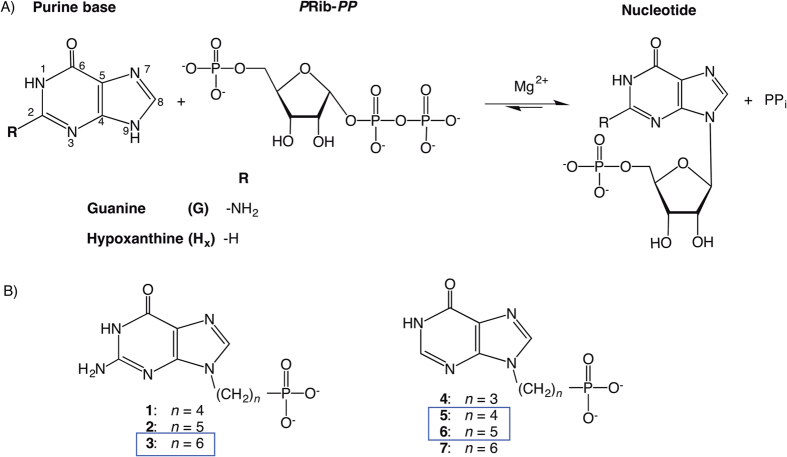
(**A**) Reaction catalyzed by HGPRT. (**B**) Structures of ANP nucleotide analogues. Inhibitors present in the crystals of *Tbr*HGPRT complexes are framed in blue.

The K_i_ values of the reaction products, GMP and IMP for *Tbr*HGPRT and for seven ANPs^19^ were determined (Table 2). The K_i_ for GMP is 2.5-fold lower than for IMP (30.5 *vs* 77.3 μM) (Table 2), values consistent with guanine being the preferred substrate (Table 1). By comparison, these values are 5- and 14-fold, respectively, higher than for human HGPRT (Table 2). The seven **ANPs** posses either guanine or hypoxanthine as the base and also vary in the number of carbon atoms connecting the N9 of the purine base to the phosphonate moiety, ranging from three to six carbon atoms (Fig. 1B). All of these compounds are competitive inhibitors of *Tbr*HGPRT. Compound **2** (with guanine as the base) and **6** (with hypoxanthine as the base) both have K_i_ values significantly lower than their corresponding nucleoside monophosphates, while the remaining five ANPs have K_i_ values similar to or greater than those for the nucleoside monophosphates. Common to **2** and **6** is the fact that they both have five carbon atoms linking the base to the phosphorus atom and this is a key factor that results in tighter binding compared to the other inhibitors. A similar observation was made for human HGPRT (Table 2). However, **2** binds more tightly to human HGPRT (0.5 μM *vs* 2.3 μM), while for **6** the reverse is true since it binds more tightly to *Tbr*HGPRT (>100 μM *vs* 15.8 μM) (Table 2).

### Quaternary structure of *Tbr*HGPRT in solution

The 6-oxopurine PRTs have been found to exist as either tetramers or dimers in solution^28^. Human HGPRT is a tetramer, while *Tcr*HGPRT can exist as either a dimer or tetramer^29^. To determine the quaternary structure of *Tbr*HGPRT in solution, a sample was analyzed by SEC-MALLS (size exclusion chromatography multi-angle laser light scattering). The results show that the major component (98%) has a molecular weight of 50.4 ± 1.7 kDa (Supplementary Figure 1B) while there is also a minor component present at a molecular weight of 106 kDa. The mass of the major component agrees well with the calculated molecular mass of 48.5 kDa for the *Tbr*HGPRT dimer including the attached His_6_-tag^30^, while the minor component may represent either the tetrameric protein or an impurity. Thus, *Tbr*HGPRT has a quaternary structure similar to that of *Tcr*HGPRT, but different to that of human HGPRT. It is well established as to why the 6-oxopurine PRTs are required to be dimeric. One of the primary factors is that the adjoining subunit holds the side-chain of a highly conserved lysine residue in place, such that it does not block the binding of *P*Rib-*PP.* In so doing its backbone atoms form a non-proline *cis* peptide bond whose amide nitrogen can bind to a phosphoryl oxygen atom of *P*Rib-*PP* or PP_i_. However, it is not clear why some 6-oxopurine PRTs are tetramers and others can exist as both, though it is speculated that tetramer formation may increase the overall stability of the enzyme^24^.

### Crystal structures of *Tbr*HGPRT

The crystal structures of *Tbr*HGPRT in complex with GMP, IMP, and compounds **3**, **5** and **6** were determined to 2.73, 2.51, 1.52, 2.89 and 2.81 Å resolution, respectively. Thus, structures of complexes with the two nucleotide products and **ANPs** where the number of atoms in the linker varies from four to six have been obtained. Data collection and refinement statistics for these five structures are presented in Supplementary Table 1. In the crystal structures with GMP and **3** a dimer is observed in the asymmetric unit. In the complex with **6** there are two separate dimers in the asymmetric unit, and in the complex with IMP or **5** two independent subunits are present in the asymmetric unit. However, these subunits do form a dimer due to pairings with crystallographic symmetry mates (Supplementary Figure 2). Thus, the crystallographic and SEC-MALLS data agree in demonstrating that *Tbr*HGPRT is dimeric.

**Figure 2:**
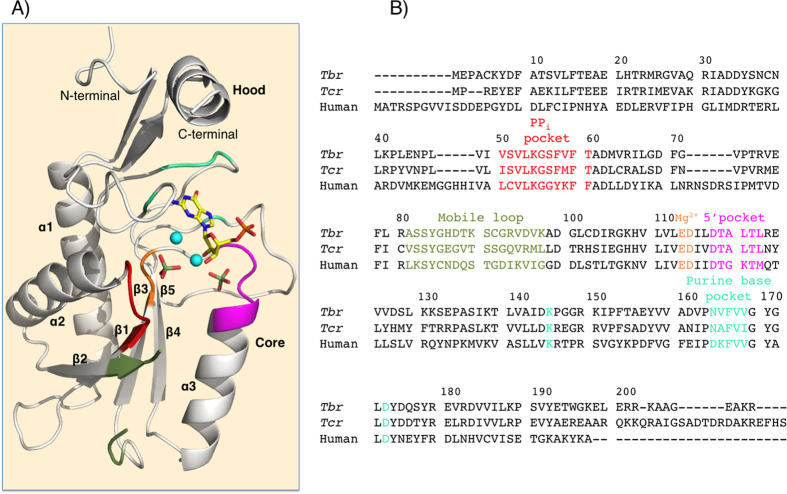
Sequence and structure of *Tbr*HGPRT. **(A**) Subunit structure of *Tbr*HGPRT in complex with GMP. Two sulfate ions and two magnesium ions (blue spheres) are also observed in the structure. (**B**) Comparison of the amino sequences of *Tbr*HGPRT*, Tcr*HGPRT and human HGPRT. The coloured regions in the sequence alignment (**B**) correlate with the coloured regions in the structural image (**A**).

Supplementary Table 1 indicates that there are three regions where the *Tbr*HGPRT polypeptide is disordered in the crystal structures. These are at both the N- and C-termini and within a region described as the large mobile loop that is also disordered in many other 6-oxopurine PRT crystal structures^10^. In *Tbr*HGPRT, the amino acid residues in this loop are located between R80 and A99 (Fig. 2). Although it is not unusual for these residues to be disordered, in the presence of ligands that resemble the transition state of the reaction, this region does become ordered and completely sequesters the active site from the solvent. Such structures have been determined for, amongst others, human HGPRT in complex with immucillinGP.PP_i_.Mg^2+ ^31, and *Tcr*HGPRT in complex with HPP.*P*Rib-*PP*.Mg^2+ ^31,32. The immucillins are transition state analogs that have a centrally located iminorbitol ring to mimic the positive charge predicted in the oxocarbenium-like transition state^31^. These compounds and PP_i_ form sufficient interactions with the large mobile loop to ensure that it is ordered and closed over the active site. HPP, 7-hydroxy [4,3-d]pyrazolopyrimidine, is a hypoxanthine analog where the N9 atom has been replaced with a carbon atom. Thus, when this molecule and *P*Rib-*PP* are bound to the enzyme at the same time, they mimic the binding of the two substrates but the transfer reaction cannot proceed. Again, in this complex there are sufficient interactions to ensure closure and ordering of this loop^32^. Since none of the five *Tbr*HGPRT structures represent the transition state, the lack of visible electron density for the mobile loop is in agreement with structural data for other 6-oxopurine PRTs whose structures are available.

### Subunit structure of *Tbr*HGPRT

Analysis of the structure of the *Tbr*HGPRT.GMP complex using the Dali server^33^ shows that it has the closest structural similarity with *Tcr*HGPRT and *L. tarentolae* HGPRT (Supplementary Table 2). The next eight proteins with the highest similarity scores are of bacterial origin. *Pf*HGXPRT has a slightly lower similarity score than the 6-oxopurine PRTs from these bacteria. However, this can be ascribed due to the fact that the structure of *Pf*HGXPRT was acquired in the presence of transition state analogs^34^,^35^ which induce conformational changes not only in the large mobile, but also in the 5′-phosphate, 6-oxopurine, PP_i_ and magnesium binding pockets. Thus, it is not unexpected that the structures of the parasitic *Pf*HGXPRT have less similarity to the *Tbr*HGPRT compared to the bacterial enzymes whose structures were also determined in the presence of nucleotide products.

Each subunit of *Tbr*HGPRT consists of two domains, which in other 6-oxopurine PRTs have previously been referred to as the core and hood^36^. The overall structure of the core domain in all five structures consists of five parallel β-strands and three α-helixes (Fig. 2A). These are highly conserved features in the 6-oxopurine PRTs^37^,^38^. However, the architecture of the hood domain in the 6-oxopurine PRTs varies significantly depending on its species of origin. In *Tbr*HGPRT, the hood domain is formed by a combination of the 15 N-terminal and 26 C-terminal residues (Fig. 2B). There are three regions of secondary structure in this domain, a β-strand (A10-L15) at the N-terminus of the polypeptide, and a β-strand (V184-L187) from the C-terminal region that combine together to form a small antiparallel β-sheet, while at the N-terminus a short α-helix (V191-L199) is visible. The remaining 10–14 residues at the N-terminus (Supplementary Table 1) are not visible in the electron density in any of the five structures.

### Comparison of the *Tbr*HGPRT and *Tcr*HGPRT structures

**Figure 3:**
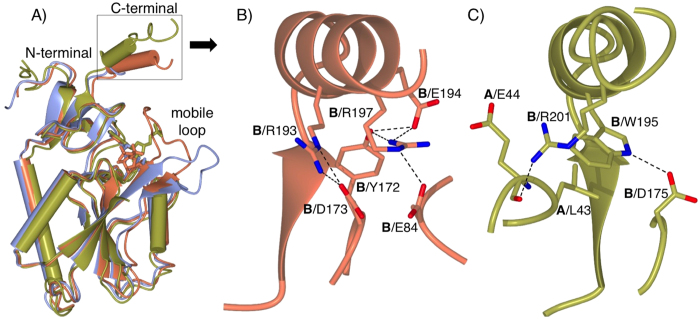
Comparison of the structures of *Tcr*HGPRT and *Tbr*HGPRT. (**A**) The*Tcr*HGPRT.*P*Rib*PP*.HPP.Mg^2+^ complex (PDB code: 1TC2) is in brown, the *Tcr*HGPRT.IMP complex (PDB code: 1P19) is in blue, and the *Tbr*HGPRT.GMP is in lime green. Note that the C-terminal region, including the helix, in the *Tcr*HGPRT.IMP is disordered. (**B**) Interactions at the C-terminal in the *Tcr*HGPRT.*P*Rib*PP*.HPP.Mg^2+^ complex (PDB code: 1TC2). (**C**) Interactions at the C-terminal in the *Tbr*HGPRT.GMP complex.

These two enzymes have 53% amino acid sequence identity and the Dali analysis shows that the RMSD value is only 0.9 Å for 166 structurally equivalent residues (Supplementary Table 2). Both of these statistics suggest a high degree of similarity between these two enzymes. Figure 3A shows that the core domains and the β-sheets in the hood domains of the two enzymes superimpose well with each other. However, the structures differ significantly at the C-terminus. The α-helix situated near the C-terminus of *Tbr*HGPRT.GMP complex (L187-W195) has no equivalent in the *Tcr*HGPRT.IMP complex, where the corresponding residues are disordered. However, in the *Tcr*HGPRT.HPP.*P*Rib-*PP*.Mg^2+^ complex, which mimics the transition state, this region does adopt a helix but in a different orientation (a rotation of ~15°) compared to that observed in the *Tbr*HGPRT.GMP complex (Fig. 3A–C). Thus, for *Tcr*HGPRT, this helix appears to be ordered only when the large loop is firmly closed (*i.e.* the enzyme is approaching or at the transition state). However, in the structures of *Tbr*HGPRT determined here, all of which represent the binding of products or product analogs, this helix is ordered. In the *Tbr*HGPRT.GMP complex, there are a number of interactions that stabilize the helix. These include two hydrogen bonds, one between the side-chain of R201 and the carbonyl oxygen of E44 (from the adjoining subunit) and between the nitrogen in the indole of W195 and the oxygen in the side chain of D175, in addition to a set of hydrophobic interactions between W195 and L43, also from the adjoining subunit (Fig. 3C). Thus, these interactions not only help to orient and lock the helix in place in *Tbr*HGPRT, they also stabilize the dimer (Fig. 3C). These interactions are a novel and potentially unique feature of *Tbr*HGPRT, as they have not been observed in other 6-oxopurine PRT structures determined to date.

### Comparison of the *Tbr*HGPRT and human HGPRT structures

Comparing the subunit structures of the *Tbr*HGPRT.GMP complex and human HGPRT.GMP complexes, their rmsd upon superimposition is 0.9 Å for 104 Cα atoms. Thus, about half of the polypeptide from the two proteins can be overlayed while the in the remainder there are some differences. Two polypeptide regions where there are insertions in the human enzyme compared to the *Tbr*HGPRT, are between residues 47 and 48 (*Tbr*HGPRT numbering) and between 71 and 72, where the human protein possesses five and six extra residues, respectively (Fig. 2B). Human HGPRT also has an extra β-strand at the N-terminus, which is not present in *Tbr*HGPRT (Fig. 2B). Additionally, the C-terminus of *Tbr*HGPRT is 13 residues longer than that of human HGPRT, though as described earlier these residues are not visible in the electron density maps of any of the structures.

The C-terminal helix in human HGPRT also adopts a different orientation compared to its position in *Tbr*HGPRT. After superimposition of the two structures, this helix is rotated by ~30°. In the human HGPRT.GMP complex two side-chains in the helix make hydrogen bonds to other residues in the enzyme. These are intra-subunit hydrogen bonds between K212 and Y194 and between Y215 and D30, and a hydrogen bond between Y215 and S91 of an adjacent subunit. In the humanHGPRT.immucillinGP.PP_i_.Mg^2+^ complex this helix does not play a role in closing the large mobile loop over the active site, and instead residues D106 and N195 function to close the gap between the hood and core domains of the protein. Thus, there is a likely difference in the way in which the mobile loops close over the active site in the human and parasite enzymes. This discriminating feature could be particularly relevant for achieving inhibitor selectivity.

### Oligomeric states of *Tbr*HGPRT and human HGPRT

The crystal structures (Supplementary Figure 3A) and SEC-MALLS (Supplementary Figure 1B) data confirm that *Tbr*HGPRT is mainly present as a dimer while human HGPRT is a tetramer (Supplementary Figure 3B). Human HGPRT forms extensive contacts across two types of interface. One of these is at the dimer interface (Supplementary Figure 3B), which is common to both *Tbr*HGPRT and human HGPRT. The alternative interface is described as the tetrameric interface (Supplementary Figure 3B). Both types of interface were analyzed using PISA^39^,^40^ which showed that the *Tbr*HGPRT dimer interface in the *Tbr*HGPRT.GMP complex is formed by seven hydrogen bonds and six ion pairs, with an interface area of 1570.6 Å^2^. For the human enzyme, the interface area is similar, 1509 Å^2^, with eleven hydrogen bonds and eight ion pairs. Although the overall interface surface areas are about equal in size in the two proteins, the residues that make contact at this surface vary considerably (Supplementary Figure 3). Indeed, only two residues at the dimer interface are identical in the two enzymes. These are L53 and K54 (*Tbr*HGPRT numbering). Six other residues at the dimer interface of the two proteins are in equivalent positions when both polypeptide sequences are aligned. However the identities of these amino acid residues are not conserved in the two proteins. In *Tbr*HGPRT a cluster of four hydrophobic residues, i.e. L53, F57, V58 and F78, are key to the stabilization of this interface. In human HGPRT, the residues equivalent to F57 and V58 are replaced by charged amino acids (*i.e.* a tyrosine and lysine, respectively), which form salt bridges to stabilize the interface. In addition to these contacts, and as described earlier, W195 and L43 in *Tbr*HGPRT form a hydrophobic interface between these subunits, a structural feature that is absent in the human enzyme. Two other residues (Q176 and R182) have no counterparts in human HGPRT, and also help to stabilize the *Tbr*HGPRT dimer by forming inter-subunit associations. Namely, the side chain of Q176 forms a bond with NZ of K54 and R182 forms a hydrogen bond with the backbone oxygen of R65.

As explained above, SEC-MALLS shows that about 2% of *Tbr*HGPRT has a mass consistent with that of a tetramer (Supplementary Figure 1B). Although only a small percentage, we considered whether the arrangement of subunits observed in the human HGPRT tetramer could also occur to make a *Tbr*HGPRT tetramer. To test this idea, dimer pairs of the *Tbr*HGPRT structure were overlayed onto the human tetramer. This analysis showed that a tetramer could form, though, according to PISA, the interface area is only 353 Å^2^, a value considered small for a subunit interface. However, it is possible that, upon interaction, the termini of the polypeptides could change conformation to allow additional interactions between the adjacent subunits. Thus, it is possible that *Tbr*HGPRT could exist as a tetramer having the same oligomeric arrangement as the human enzyme, though under the (buffer) conditions used for purification and crystallization, the dimer is clearly the dominant oligomeric form of *Tbr*HGPRT.

### Active site of *Tbr*HGPRT when IMP or GMP is bound

#### Purine base binding

**Figure 4:**
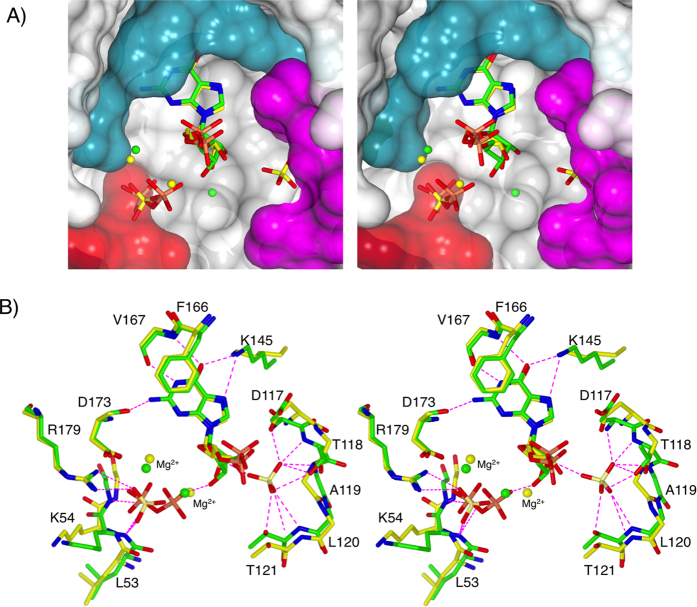
Stereoimages of the comparison of the active sites of the *Tbr*HGPRT.GMP and *Mt*HGPRT.GMP.PP_i_ complexes. (**A**) Connolly surface for the polypeptide of the *Tbr*HGPRT.GMP complex with the ligands from the *Tbr*HGPRT.GMP complex (green carbon atoms) and the *Mt*HGPRT.GMP.PP_i_ complex (yellow carbon atoms) overlayed. (**B**) Superimposition of the *Tbr*HGPRT.GMP complex (green carbon atoms) and the *Mt*HGPRT.GMP.PP_i_ complex (yellow carbon atoms).

The purine bases of IMP and GMP are held in place by a π-π stacking arrangement with F166 as well as by hydrogen bonds from the 6-oxo and N7 atoms of the purine base to the NZ atom of K145, and between the N1 purine nitrogen and the backbone carbonyl oxygen of V167. The 6-oxo atom also forms a hydrogen bond with the backbone amide of V167 (Fig. 4). In the GMP complex, the amino group of the guanine base makes an additional hydrogen bond with the main chain carbonyl of D173 (Supplementary Figure 4A). Since the guanine and hypoxanthine bases superimpose without requiring any additional movement (*i.e.* rotation and or translation) in the active site (Supplementary Figure 4C) the interaction with D173 appears to be significant to account for the 2.5-fold lower K_i_ value for GMP compared with IMP (Table 2). The only other difference is that, in the presence of GMP, a water molecule makes a hydrogen bond with the N3 atom of the purine base and coordinates with a magnesium ion (Supplementary Figure 4A), whereas when IMP is bound, this water makes a hydrogen bond only with the backbone oxygen of D173 (Supplementary Figure 4B).

#### 5′-Phosphate and pyrophosphate binding sites

Based on amino acid sequence comparison and other X-ray structures of 6-oxopurine PRTs, residues D117-L122 are predicted to form the 5′-phosphate binding pocket^36^,^41^,^42^,^43^,^44^,^45^. However, in these structures with IMP or GMP bound in the active site of *Tbr*HGPRT, the 5′-phosphate moiety does not occupy this pocket. Instead, sulphate ions were allocated to the electron density in this site in the two structures (Fig. 4 and Supplementary Figure 4). This assignment was based on the presence of 0.2 M lithium sulphate in the crystallization buffer and the fact that sulphate is a weak inhibitor of *Tbr*HGPRT (in our standard assay 25% inhibition is observed when 2M ammonium sulphate is added to the cuvette). Thus, in both the GMP and IMP complexes, the ribose ring and 5′-phosphate group are not found where they are expected to be located. Instead, the 5′-phosphate group projects outward from the active site (Fig. 4). It could be argued that, in these structures, the 5′-phosphate groups have been displaced by the sulphate ions to give this unusual binding mode, a condition that might have arisen during crystallization. However, the same binding mode has also been observed in the *M. tuberculosis* (*Mt*) HGPRT.GMP complex (PDB code: 4RHT) (Fig. 4). This structure, obtained in the absence of sulphate or phosphate, has an empty 5′-phosphate binding pocket, although the PP_i_ binding site is occupied by a PP_i_ molecule. In the *Tbr*HGPRT and the *Mt*HGPRT structures, the location and conformation of the ribose ring and positions of the 5′-phosphate groups are virtually identical (Fig. 4). Thus, these new structures of *Tbr*HGPRT provide further evidence that there is an alternate binding mode for the nucleotide products, where they are held in place largely by interactions between the enzyme and the purine base. In *Mt*HGPRT it has been proposed that this conformation could represent the nucleotide product as it is about to enter or exit the active site (Fig. 4)^24^. Thus, this also could be the case in *Tbr*HGPRT.

In addition to the sulphate in the 5′-phosphate binding pocket, a second sulphate ion is located where one of the phosphate groups of PP_i_ would be expected to bind (Fig. 4). One of the sulphate oxygen atoms forms a hydrogen bond with the side-chain of R179, while the other sulphate oxygen atoms form hydrogen bonds with the main chain amide of K54 and G55. A third sulphate oxygen also forms a hydrogen bond to the main chain amide of K54. With these interactions in place, the peptide bond between L53 and K54 is in the *cis* conformation (Supplementary Figure 4). This *cis* conformation has only ever been observed in human HGPRT when *P*Rib-*PP*^32^, PP_i_^24^,^41^ or sulfate^22^ is bound in this pocket. In the absence of stabilization by a hydrogen bonding partner, this peptide bond is expected to be in the *trans* conformation.

### Crystal structures with the three acyclic nucleoside phosphonates (ANPs)

Crystal structures of three ANPs^19^, **3**, **6** and **5**, in complex with *Tbr*HGPRT have been determined (Supplementary Table 1). The electron density for **3**, which adopts two conformations of equal occupancy, is shown in Supplementary Figure 5. The overall fold of the enzyme in the three inhibitor complexes is similar to that when GMP or IMP bind. However, when the ANPs bind, the phosphonate group occupies the predicted 5′-phosphate binding pocket. This is in contrast to the structures when the nucleotides are bound and the 5′-phosphate group projects outward from the active site (Supplementary Figure 4). The different binding conformations between the ANPs and GMP or IMP may be due to the presence of the ribose ring in the nucleotide products, bearing in mind that the goal of the enzyme is to release the reaction products as efficiently as possible. The absence of the ribose ring in the ANPs may preclude this alternate binding mode.

**Figure 5:**
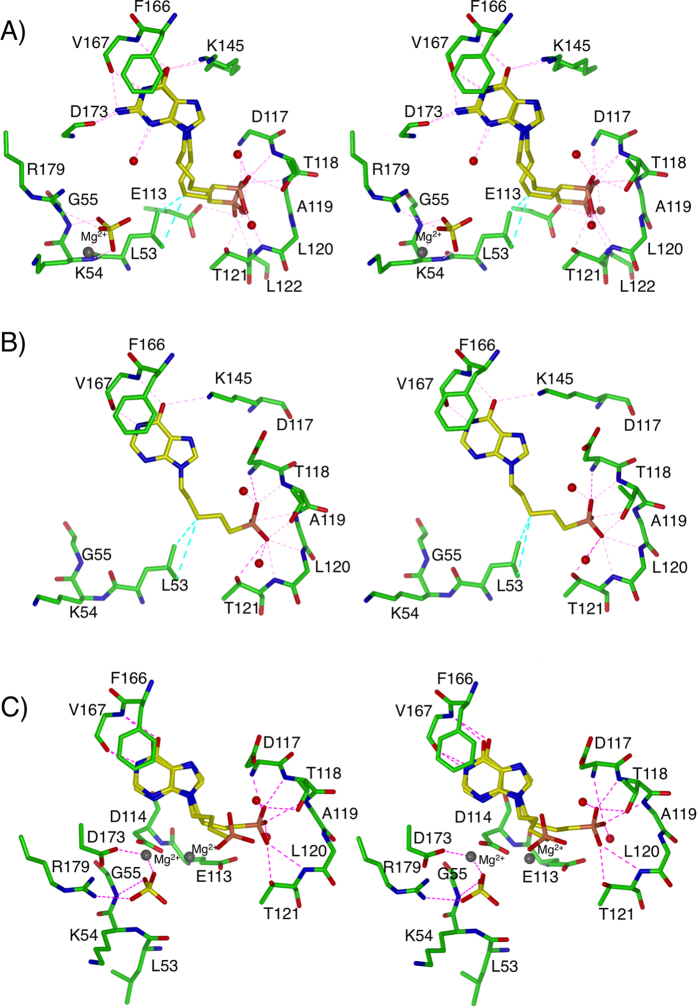
Stereoview of the active site of *Tbr*HGPRT in complex with the three ANPs. (**A**) 3, (**B**) 6 and (**C**) 5.

**Figure 6:**
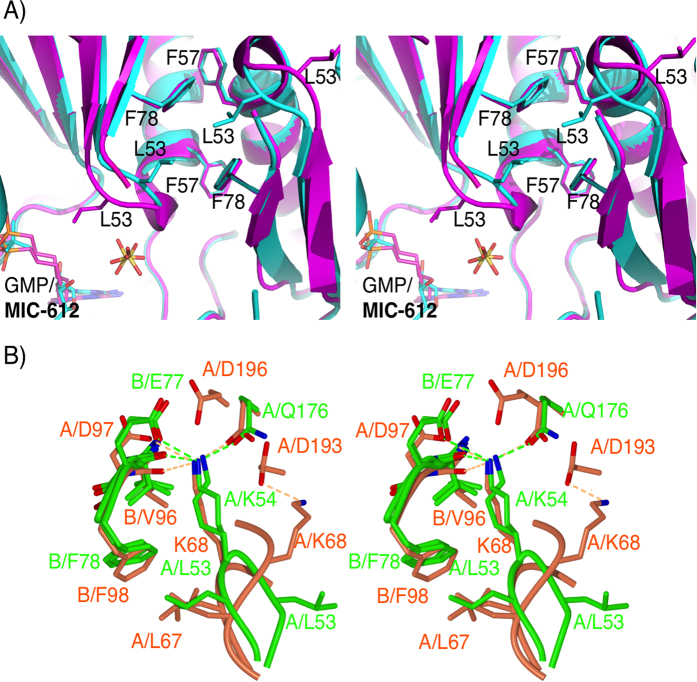
Stereoview of the *cis* to *trans* isomerization of the PP_i_ binding loop in *Tbr*HGPRT and human HGPRT. (**A**) The L53-K54 peptide bond in the *cis* conformation in the *Tbr*HGPRT.GMP complex (light blue) and in the *trans* conformation in *Tbr*HGPRT.**3** complex (magenta). (**B**) A comparison of the top of the PP_i_ binding loop in *Tbr*HGPRT (green) and human HGPRT (brown) as the L53-K54 peptide bond switches from the *trans* to the *cis* conformation.

In the *Tbr*HGPRT.**3** complex, **3** adopts two conformations resulting in the phosphonate group occupying alternative positions in the 5′-phosphate binding site (Supplementary Figure 5 and 5A). In one of these, the oxygen atoms of the phosphonate moiety form hydrogen bonds with the amide nitrogen atoms of D117 and L120, the amide nitrogen and side chain hydroxyl of T118 and the side chain hydroxyl of T121. In the second conformation, the phosphonate moiety is translated by ~1.8 Å, resulting in the formation of additional hydrogen bonds to the side-chain of E113 and the amide nitrogen of T121, and the removal of hydrogen bonds to the side chain hydroxyl and the amide nitrogen of T118. Three ordered water molecules are also found in or near this pocket. One forms a hydrogen bond with the amide nitrogen of T121 and a second forms a hydrogen bond with the side-chain of D117, thus making indirect links between the enzyme and the inhibitor, while the third water molecule makes networks with the surrounding ordered water molecules (Fig. 5A). As with GMP, the guanine base of **3** makes a π-π stacking interaction with the aromatic ring of F166, and forms hydrogen bonds to K145, the carbonyl oxygen of V167 and to the backbone carbonyl of D173 (Fig. 5A). A sulphate ion is also present in this structure where the oxygen atoms form interactions with K54 and G55 (Fig. 5A). In this structure, the bond between L53 and K54 is in the *trans* conformation and the side-chain of L53 is in hydrophobic contact with the carbon atoms of the linker in this ANP. This contrasts to the GMP or IMP complexes in which the L53-K54 peptide bond is in the *cis* conformation, and the side-chain of L53 points away from the active site and interacts with F57 from the same subunit and L53 and F78 from the adjacent subunit at the dimer interface (Fig. 6A).

The binding of **6** (with five carbons in the linker and hypoxanthine as the base) is similar to that observed for **3** (six carbons in the linker and guanine as the base) in that the purine base binds in the purine binding pocket and the phosphonate moiety binds to the amino acid residues surrounding the 5′-phosphate binding site (residues D117-T121). There are however some differences. Compound **6**, with hypoxanthine as the base cannot form a hydrogen bond between the exocyclic amino group and the main chain carbonyl of D173 (Fig. 5A,B). By comparison with **2** (also five carbons in the linker but guanine as the base) the K_i_ value is seven fold higher (15.8 μM *vs* 2.3 μM). Thus again, as observed in the IMP and GMP comparison, the guanine base confers stronger associations than hypoxanthine to *Tbr*HGPRT. Compared to **3,** the purine ring of **6** is translated by ~1 Å, however four hydrogen bonds (two to K145 and two to V167) that hold the purine ring in place are conserved in the two complexes (Fig. 5B). The phosphonyl oxygen atoms in **6** form hydrogen bonds with the amide nitrogen atoms of D117, T118, A119, L120 and the side-chains of T118 and T121 (Fig. 5B) similar to the binding of one of the conformations of **3**. Compound **6** with one fewer carbon atom than **3**, is able to reach into the 5′-phosphate binding site with its phosphonate group, thus explaining the similar K_i_ values for **3** and **6** (15.8 μM *vs* 21.2 μM). However, when the base is hypoxanthine and the linker is six carbon atoms (**7**) there is a marked increase in the K_i_ value (>100 μM). Thus, this combination is not favorable for tight binding.

In the *Tbr*HGPRT**.5** complex (hypoxanthine as the base and four carbon atoms in the linker) there are two different subunits in the asymmetric unit. In one subunit, **5** adopts two alternative conformations in the active site. One of these allows the phosphonate oxygen atoms to fit into the 5′-phosphate binding pocket, interacting with the amide nitrogen atoms of D117, T118 and L120 and the side-chain oxygen atoms of T118 and T121 (Fig. 5C), while in the other conformation the phosphonate group protrudes out of the active site and away from the 5′-phosphate binding pocket. In the second subunit only this latter conformation is present.

One of the most important interactions between a 6-oxopurine PRT and the nucleoside monophosphate is the hydrogen bond between the 6-oxo group and a highly conserved lysine side-chain (K145 in *Tbr*HGPRT (Fig. 2B)). This bond is critical in discriminating the 6-oxopurine from a 6-aminopurine (such as adenine). However, in the complex with **5** there is no such association with K145 located away from the purine base and the base itself rotated slightly compared to when GMP is bound, such that the distance between the 6-oxo group and this lysine is 3.9 Å at its closest approach (Fig. 5C). A sulphate ion is also observed in the active site, and is located in the PP_i_ binding pocket where it interacts with the amide nitrogen of G55, the side-chain of R179 and one magnesium ion. A second magnesium is located between E113 and D114, which is a Mg^2+^ binding site in the 6-oxopurine PRTs (Fig. 2B)^24^,^31^,^34^,^46^. In this complex, the peptide bond between L53 and K54 is in the *cis* conformation as observed when GMP and IMP bind (Fig. 5C), but contrasts to that observed for **3** and **6** where it is in the *trans* conformation (Supplementary Figure 4). Again, in this complex, a sulphate ion is present in the PP_i_ binding site and this is the common factor in stabilizing the *cis* peptide bond conformation in all three structures. The structure of the complex with **5** suggests that the shorter linker is not long enough to promote favourable hydrophobic interactions with the L53 side-chain, thus keeping the L53-K54 peptide bond in the *cis* conformation, resulting in the L53 side-chain to point away from the active site.

All of the compounds in Table 2 were subjected to crystallization trials with *Tbr*HGPRT. Along with the three structures described above, crystals with **2** were also obtained and data were collected to 2.5 Å resolution. However, due to twinning of the crystals, the structure could not be refined for deposition to the protein data bank. Nevertheless, it was clear from the electron density maps (Supplementary Figure 6A) that **2** does make similar interactions to *Tbr*HGPRT as **6**, but with an extra interaction between the N2 of the purine base and the side chain of D173 (Supplementary Figure 6B), explaining the lower K_i_ value.

In summary, the results from the inhibition and crystallographic studies show that ANPs that have five carbon atoms in the linker (*e.g.* **6** and **2**) have the best potency for *Tbr*HGPRT and the reason for this is that both the purine binding pocket and 5′-phosphate binding pocket are fully occupied by this specific type of ANPs. Compounds that contain six carbon atoms (*e.g.* **3**) can also fill both sites, but the linker adopts a slightly different conformation which appears less favorable for binding (Supplementary Figures 4–5 and Fig. 5). Linkers with both five and six carbon atoms can induce van der Waals associations with the side-chain of L53. Compounds that contain four carbon atoms can also occupy both the purine base and 5′-phosphate binding pockets but are too short to make as many stabilizing bonds compared to when the linker is five or six carbon atoms in length, hence the higher K_i_ values. The crystal structures show that the ANPs with four carbon atoms can adopt multiple conformations and that the phosphonate group does not fully insert into the 5′-phosphate binding pocket.

### Differences between the PP_i_ binding loops in human HGPRT and *Tbr*HGPRT

**Figure 7:**
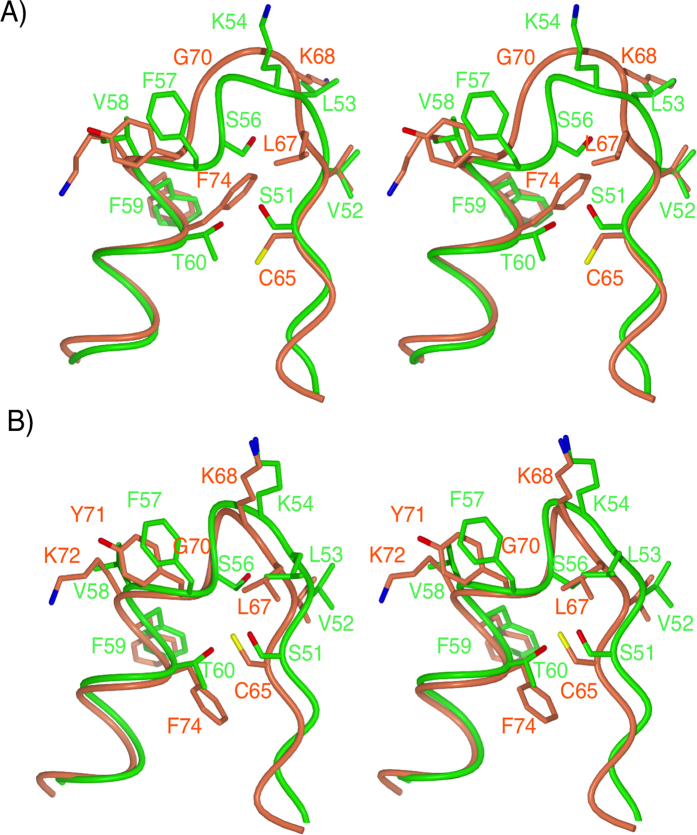
Stereoview of the superimposition of the PP_i_ binding loops in human HGPRT and *Tbr*HGPRT. (**A**) The *Tbr*HGPRT.**6** complex (green carbon atoms) and unliganded human HGPRT (PDB code: 1Z7G) (orange carbon atoms) The L53-K54 (*Tbr*HGPRT) and L67-K68 (human HGPRT) peptide bonds are in the *trans* conformation in these structures. (**B**) In the *Tbr*HGPRT.GMP complex (green carbon atoms) and human HGPRT.immucillinGP.PP_i_.Mg^2+^ (PDB code: 1BZY) (orange carbon atoms) the L53-K54 (*Tbr*HGPRT) and L67-K68 (human HGPRT) peptide bonds are in the *cis* conformation.

In crystal structures of human HGPRT, the peptide bond between L67 and K68 can exist in either a *trans* or *cis* conformation. When this conformational change occurs, the side chain of K68 rotates by 180° out of the active site to make room for PP_i_ or other ligands containing groups capable of mimicking all or part of PP_i_^31^,^46^. Thus, this process plays a critical function in the catalytic cycle of these enzymes, opening the active site so that substrates have access for binding. In the complex with **5**, the closest approach to the amide nitrogen of the *cis* peptide bond is 3.7 Å from the oxygen of the sulphate ion. Thus, for this enzyme, a relatively long distance ionic interaction is all that appears to be required to favour the *cis* conformation over the *trans* conformation. It is unclear whether this means that, in *Tbr*HGPRT, the peptide bond remains in the *cis* conformation throughout the catalytic cycle or whether in the absence of this sulphate ion, the bond would revert to the *trans* conformation. It is also unclear whether or not the binding of **3** or **6** is sufficient to rotate this bond from *cis* to *trans*. By either of these scenarios, the mechanism by which the PP_i_ binding site becomes ready for substrate binding appears to differ between the human and *Tbr* enzymes. There is little resemblance in the amino acid residues that constitute the PP_i_ binding loop of these two enzymes. In the human enzyme, the loop consists of the sequence -64-LCVLKGGYKFF-74- whereas in *Tbr*HGPRT this sequence is −50-VSVLKGSFVFT-60- (Fig. 2B). These sequence differences carry through to differences in their three-dimensional structures, especially when the L67-K68/L53-K54 peptide bond (human/*Tbr* HGPRT numbering) is in the *trans* conformation in the two enzymes (Figs 6 and 7A). In the human enzyme when in the *trans* conformation (*e.g.* the unliganded enzyme), the side-chain of L67 lies across the PP_i_ loop where it is held in place by hydrophobic interactions with the side-chain of F74 (Fig. 7A). On the other hand, in *Tbr*HGPRT the equivalent residue, L53, protrudes into the active site and makes no contact with the rest of the PP_i_ loop (Fig. 7A). In effect, L53 pushes between E113 and D114, either displacing or preventing magnesium from binding (Figs 2B and 5). The result of this difference is that the side-chains of K68 in the human enzyme and L53 in *Tbr*HGPRT have similar orientations when the peptide bond of both enzymes is in the *trans* conformation (Figs 6B and 7A). Figure 7A also clearly demonstrates that the backbone around the G70 (human numbering) adopts a very different conformation in the human and *Tbr* enzymes, when both enzymes have the L67-K68 peptide bond in the *trans* conformation. However, when the L53-K54 bond (*Tbr* numbering) is in the *cis* conformation (Fig. 7B) the side chain of L53 fits neatly in a pocket surrounded by three hydrophobic residues, two of which belong to the adjacent subunit of the dimer (Fig. 6A). K54 in *Tbr*HGPRT also adopts an orientation such that its side-chain is positioned away from the active site and is in an extended conformation held in place by a hydrogen bond to the carbonyl of V76, an ionic bond to the side-chain of E77, a hydrogen bond to the side-chain amide of Q176 and hydrophobic interactions to the carbon atoms of K54 and the aromatic ring of F78 (Fig. 6B). In *Tbr*HGPRT there is a serine at position 56, which makes a hydrogen bond with the main chain carbonyl oxygen of V52 and a hydrogen bond is also formed between the side-chains of S51 and T60 (Fig. 7A). These two stabilizing bonds firmly lock the bottom of this loop in place regardless of whether or not the L53-K54 bond is in the *cis* or *trans* conformation (Fig. 7A,B). In human HGPRT, the three equivalent residues to S56, S51 and T60 are G70, C65 and F74, with the only interaction being a hydrophobic contact between C65 and F74. Thus, in this region, in the human enzyme there are very few non-covalent restraints.

In summary, when the L67-K68 peptide bond switches from *trans* to *cis* in human HGPRT multiple changes occur within the PP_i_ loop. These include changes to the backbone dihedral angles throughout the entire loop and to the orientation of the side-chains, especially F74 and C65 (Fig. 7A,B). On the other hand in *Tbr*HGPRT, there are only two changes to the polypeptide that occur to accompany the *trans* to *cis* peptide conformation change. These are a 130° rotation in the backbone phi dihedral angle of L53 and a 50° rotation in the backbone psi dihedral angle of K54.

## Conclusions

The 6-oxopurine PRT from *T. brucei* studied here utilizes both guanine and hypoxanthine with similar k_cat_ values, though the K_m_ value for hypoxanthine is 2-fold higher than for guanine. For the *Tbr*HGPRT used in this study, the ANP with guanine as the purine base and five carbon atoms in the linker between the purine and phosphonate moiety is a good inhibitor with a K_i_ value of 2.3 μM. The design of improved inhibitors of *Tbr*HGPRT should be based on this compound as a lead. It is clear that there are several differences in the properties of human and *T. brucei* enzymes. These include their quaternary structures, the composition and folding of the PP_i_ binding loop, the structures of the hood domains and their overall flexibility. These differences have the potential to be exploited in order to achieve selective inhibition of *Tbr*HGPRT over human HGPRT.

## Materials Methods

### Cloning and overexpression of TbrHGPRT

The DNA sequence for *Tbr*HGPRT (GenBank accession no. AAA16328.1) along with the DNA sequence coding for a His_6_-tag attached to the N-terminus was cloned into the pET-22b expression vector by GeneArt^®^. The plasmid containing this gene was then used to transform competent BL-21 (DE3) *E. coli* cells. Cell colonies containing the construct of interest were selected for growth on agar LB plates supplemented with ampicillin and chloramphenicol. Cells were grown at 37 °C in LB media. Induction of expression was at 30 °C for 8 hours upon the addition of 0.5 M isopropyl β-D-1-thiogalactopyranoside (IPTG). Cells containing the expressed protein were centrifuged at 4 °C. The cell pellet was re-suspended and stored in 0.1 M Tris-HCl, 0.012 M MgCl_2_, 1 mM PMSF, 0.2 mM *P*Rib*PP*, 1 mM dithiothreitol (DTT), pH 7.4 at −80 °C. Cells were lysed by the addition of lysozyme (2–4 mg per ml of cells) in the presence of protease inhibitor cocktail Roche^®^ (1 tablet per 50 mL of cells) and DNase 50 μg/ml of cells in buffer. The cells were kept on ice for 1 hour followed by three freeze-thaw steps in liquid nitrogen. The soluble fraction was separated by centrifugation at 4 °C. The recombinant enzyme was purified by Ni^2+^ chromatography column (Profinity^TM^ IMAC resin) at 4 °C. The elution buffer was 0.1 M Tris-HCl, 0.012 M MgCl_2_, 0.3 M NaCl, 0.3 M imidazole, 1 mM DTT, pH 7.4. The eluent was then dialyzed into 0.03 M phosphate buffer, 0.012 M MgCl_2_ and 1 mM of DTT, pH 7.4 at 4 °C overnight. Some enzyme was aliquoted and stored for kinetic studies at 1.20 mg/mL. For X-ray crystallographic studies, the remainder was concentrated to 50 mg/mL using an Amicon concentrator (model 12–76 psi) and an Amicon Ultra-15 centrifugal device. All of the samples were subsequently stored at −80 °C. Sample purity was assessed by (12%) SDS-PAGE. The Direct Detect™ was used to measure the concentration of all of the protein samples. This method measures the absorbance of the amide bonds in the polypeptide at A_220_.

### Multi-angle laser light scattering

For SEC-MALLS, a DAWN HELEOS II 18-angle light scattering detector coupled with an Optilab T-rEX refractive index detector (Wyatt Technology, Santa Barbara, CA) was connected in-line with a Superdex 200 10/300 gel filtration column (GE Healthcare). After equilibrating the column with 0.03 M phosphate, 0.012 M MgCl_2_ and 1 mM of DTT, pH 7.4, the protein sample was injected onto the column and chromatographic separations were performed at room temperature at a flow rate of 0.4 mL/min. The program Astra 5.3 was used to calculate the molecular mass of the protein^47^,^48^.

### Enzyme activity and determination of kinetic constants

Enzyme activities were measured in 0.03 M phosphate, 0.012 M MgCl_2_ and 1 mM DTT, pH 7.4, in a continuous spectrophotometric assay at 25 °C. Under these conditions, the ∆ε values for conversion of hypoxanthine to IMP, guanine to GMP and xanthine to XMP at 245, 257.5, 255 nm are 2439 M^−1^ cm^−1^, 5817 M^−1^ cm^−1^ and 4685 M^−1^ cm^−1^, respectively. One unit of activity is defined as the number of μmol of purine base converted to its mononucleotide product per minute.

The *K*_*m*_ for *P*Rib-*PP* was measured by varying its concentration from 20 to 700 μM in the assay in the presence of 40 μM guanine using 3 nM enzyme in the assay. The *K*_*m*_ values for guanine, hypoxanthine and xanthine were measured by varying their concentrations from 2 to 56 μM, 3 to 56 μM, and 9 to 70 μM respectively in the presence of 500 μM *P*Rib-*PP*. The kinetic constants were calculated using GraphPad Prism 6.0.

### Inhibition studies

The synthesis and characterization of 9-substituted phosphonoalkyl purines was previously reported^19^. The *K*_*i*_ values were determined in 0.03 M phosphate, 0.012 M MgCl_2_ and 1 mM DTT, pH 7.4 at 25 °C. The concentration of guanine was fixed at 40 μM, and the concentration of *P*Rib-*PP* varied from 20 to 630 μM. The concentration of the enzyme was 2 nM. The concentration of inhibitor in the cuvette was in the range of 4–120 μM. The *K*_*mapp*_values were calculated using GraphPad Prism 6.0, and the *K*_*i*_ values were calculated using the formula 
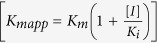
.

### Crystallization and structure determination

Initial experiments for the screening of crystallization conditions was performed using the hanging drop method vapor-diffusion and incomplete factorial screens INDEX HT™ (Hampton research) JCSG-*plus* HT-96, PACT *premier*^TM^, ProPlex (Molecular dimensions). These studies provided the leads for optimization. All crystals for diffraction experiments were subsequently obtained by mixing 1 μL of the *Tbr*HGPRT ligand complex and 1 μL of well solution. The protein-ligand complexes was prepared using 36 mg/mL, 28 mg/mL, 60 mg/mL and 28 mg/mL of enzyme in the presence of 3.2 mM (GMP), 3.2 mM (IMP), 4.6 mM (**6**), 4.6 mM (**5**) and 1.8 mM (**3**), respectively followed by incubation on ice for 10 min. The reservoir solution for the complexes with GMP (Supplementary Figure 1C), IMP and **5** and **3** was 25% PEG 3350, 0.2 M lithium sulfate and 0.1 M Bis-Tris all within a pH range of 5 to 5.5. For **6** the well solution was 25% PEG 3350, 0.2 M sodium iodide, 0.1 M Bis-Tris propane, pH 6.5. Prior to cryocooling, all crystals were transferred to a cryoprotectant solution containing reservoir solution, 20% glycerol and 1–5 mM ligand. Crystals were subsequently transported to the Australian Synchrotron and robotically placed into the cryostream (100 K) on beamline MX1. X-ray data were collected remotely using BLU-ICE^49^. Data were, integrated, scaled and merged using XDS^50^. The structure of the complex with **3** was solved by molecular replacement using PHASER^51^ and the structure of *Tcr*HGPRT (PDB code 1TC2) as the starting model. The starting models for the complexes with GMP, IMP, **5** and **6** were based upon the refined model of the complex with **3**. Refinement and model building was performed with PHENIX 1.7.3^52^ and COOT 0.8.2^53^.

## Additional Information

**How to cite this article**: Terán, D. *et al*. Crystal structures and inhibition of *Trypanosoma brucei* hypoxanthine–guanine phosphoribosyltransferase. *Sci. Rep.* **6**, 35894; doi: 10.1038/srep35894 (2016).

**Publisher’s note:** Springer Nature remains neutral with regard to jurisdictional claims in published maps and institutional affiliations.

## Supplementary Material

Supplementary Information

## Figures and Tables

**Table 1:** Kinetic constants for the naturally occurring substrates of *Tbr*HGPRT, *Tcr*HGPRT and human HGPRT.

Substrate	K_m_ (μM)	k_cat_ (s^−1^)	k_cat_/K_m_ (μM^−1^s^−1^)
*Tbr*HGPRT			
Guanine^a^	2.3 ± 0.4	23.8 ± 0.3	10.3
Hypoxanthine^a^	5.5 ± 0.3	17.1 ± 0.3	3.1
Xanthine^a^	Weak activity		
*P*Rib-*PP*^a^	103 ± 16		
*Tcr*HGPRT^b^,^c^			
Guanine	9.9 ± 0.29	41.2 ± 2.9	4.2
Hypoxanthine	8.6 ± 1.0	23	2.7
Xanthine	No activity reported		
*P*Rib-*PP*	32.4 ± 3.1		
Human HGPRT^d^			
Guanine	1.9 ± 0.4	8.2 ± 0.6	4.3
Hypoxanthine	3.4 ± 1.0	5.2 ± 0.4	1.5
Xanthine	Not a substrate		
*P*Rib-*PP*	59.6 ± 6		

^a^0.03 M phosphate buffer, 0.012 M MgCl_2_ and 1 mM of DTT, pH 7.4.

^b^(Canyuk *et al*.)^43^.

^c^(Allen & Ullman)^54^.

^d^(de Jersey *et al*.)^10^.

**Table 2:** K_i_ values (μM) of the nucleotide products and the ANPs for the PRTases.

Compound	Structure	*Tbr*HGPRT^a^	Human HGPRT^b^,^c^
GMP	G-ribose-PO_3_^2−^	30.5 ± 1.5	5.8 ± 0.2
1	G-[CH_2_]_4_-PO_3_^2−^	23.7 ± 0.3	21
2	G-[CH_2_]_5_-PO_3_^2−^	2.3 ± 0.1	0.5
3	G-[CH_2_]_6_-PO_3_^2−^	21.3 ± 0.4	0.5
IMP	Hx-ribose-PO_3_^2−^	77.3 ± 5.4	5.4 ± 1.2
4	Hx-[CH_2_]_3_-PO_3_^2−^	>100	>100
5	Hx-[CH_2_]_4_-PO_3_^2−^	58.6 ± 4	>100
6	Hx-[CH_2_]_5_-PO_3_^2−^	15.8 ± 1.8	>100
7	Hx-[CH_2_]_6_-PO_3_^2−^	>100	77

^a^0.03 M phosphate, 0.012 M MgCl_2_ and 1 mM of DTT, pH 7.4.

^b^(Cesnek *et al*.)^19^.

^c^(Keough *et al*.)^18^. G = guanine. Hx = hypoxanthine.
