# Supplementary material for: Crystal structures and inhibition of Trypanosoma brucei hypoxanthine–guanine phosphoribosyltransferase
Source: Sci Rep. 2016 Oct 27;6:35894. doi: 10.1038/srep35894 (PMC5081515; doi:10.1038/srep35894)
Supplement: Supplementary Information [file srep35894-s1.pdf]

SUPPLEMENTARY INFORMATION

Crystal structures and inhibition of *Trypanosoma brucei* hypoxanthine – guanine phosphoribosyltransferase

David Terán, Dana Hocková, Michal Česnek, Alena Zíková, Lieve Naesens, Dianne T. Keough and Luke W. Guddat

Supplementary Table 1. Data collection and refinement statistics for the *Tbr*HGPRT complexes.

Complex	**GMP**	**IMP**	**5**	**6**	**3**
Crystal parameters					
a, b, c (Å)	93.94, 111.71, 45.23	94.05, 109.82, 44.29	45.11, 93.89, 109.78	57.09, 88.63, 94.66	94.38, 108.79, 44.65
α, β, γ (°)	90, 90, 90	90, 90, 90	90, 90, 90	90, 107.16, 90	90, 90, 90
Space group	$P\,2_1\,2_1\,2$	$P\,2_1\,2_1\,2$	$P\,2\,2\,2_1$	$P\,2_1$	$P\,2\,2\,2_1$
Crystal size (mm)	0.3 x 0.1 x 0.05	0.3 x 0.3 x 0.1	0.3 x 0.1 x 0.05	0.3 x 0.3 x 0.1	0.3 x 0.3 x 0.1
Diffraction data					
Resolution range (Å)	48.01-2.73	47.42-2.48	47.39-2.81	46.46-2.96	47.19-1.50
Observations	92871 (12399)	124032 (13582)	84731 (12137)	63510 (10051)	533518 (23061)
Unique reflections	13262 (1714)	17272 (1896)	11686 (1642)	18905 (2988)[a]	73902 (3502)
Completeness (%)	99.9 (99.2)	99.8 (98.9)	98.8 (97.4)	99.6 (98.5)	99.8 (96.2)
R_{merge}	0.161 (0.76)	0.079 (0.807)	0.105 (0.712)	0.142 (0.539)	0.112 (0.891)
$R_{p.i.m.}$	0.07 (0.327)	0.034 (0.348)	0.061 (0.415)	0.142 (0.532)	0.048 (0.411)

$\langle I \rangle / \langle \sigma(I) \rangle$	8.6 (1.9)	14 (1.9)	13 (2.6)	5.9 (1.6)	11.1 (2)
Refinement					
Resolution limits (Å)	2.7	2.5	2.8	2.9	1.5
R_{work}	0.236	0.252	0.274	0.246	0.161
R_{free}	0.272	0.284	0.309	0.294	0.178
RMSD bond lengths (Å)	0.002	0.001	0.002	0.002	0.016
RMSD angles (^{o})	0.558	0.474	0.499	0.468	1.5
Components of the asymmetric unit					
Dimers	1	1	1	2	1
Visible amino acids	A/(-)1-80,101-199<br>B/(-)1-80,103-199	A/11-80,105-192<br>C/5-80,104-199	A/11-80,104-192<br>B/7-80,102-197	A/7-79,104-197<br>B/7-79,102-195<br>C/7-79,105-192<br>D/5-79, 103-196	A/5-80,103-199<br>B/5-80,100-196
Ligands	2xGMP, 4xSO_4^{2-}	2xIMP, 4xSO_4^{2-}	2x**5**, 3xSO_4^{2-}	4x**6**	2x**3**, 2xSO_4^{2-}
Mg^{2+}	4	5	6	0	2
Waters	85	48	13	78	493

Ramachandran plot (%)					
Favoured	96.0	92.4	93.8	91.8	96.4
Outliers	0.3	1.2	1.2	1.4	0.3

[a]Values in parentheses are for the outer resolution shell.

Supplementary Table 2. Results from Dali analysis using the *Tbr*HGPRT.GMP as the search model.

	ORGANISM	PDB	Z	RMSD	LALI	NRES	%ID
**1**	*T. cruzi*	1TC1	27.5	0.9	166	186	58
**2**	*L. tarentolae*	1PZM	26.3	1.0	163	170	62
**3**	*M. tuberculosis*	4RHX	23	2.4	164	175	37
**4**	*T. tengcongensis*	1YFZ	22.5	2.3	163	178	36
**5**	*B. anthracis*	3O7M	22.5	1.4	151	164	35
**6**	*S. aureus*	4RQA	22.4	1.4	157	176	35
**7**	*S. typhimurium*	1J7J	22.4	1.4	152	163	38
**8**	*V. colerae*	3OHP	22.2	1.4	153	166	38
**9**	*E. coli*	1G9S	22.2	1.4	154	169	36
**10**	*B. faecium*	4PFQ	21.7	1.5	157	177	32

*Z represents the similarity with other organisms. RMSD root mean square standard deviation for the Cα atoms after superimposition.

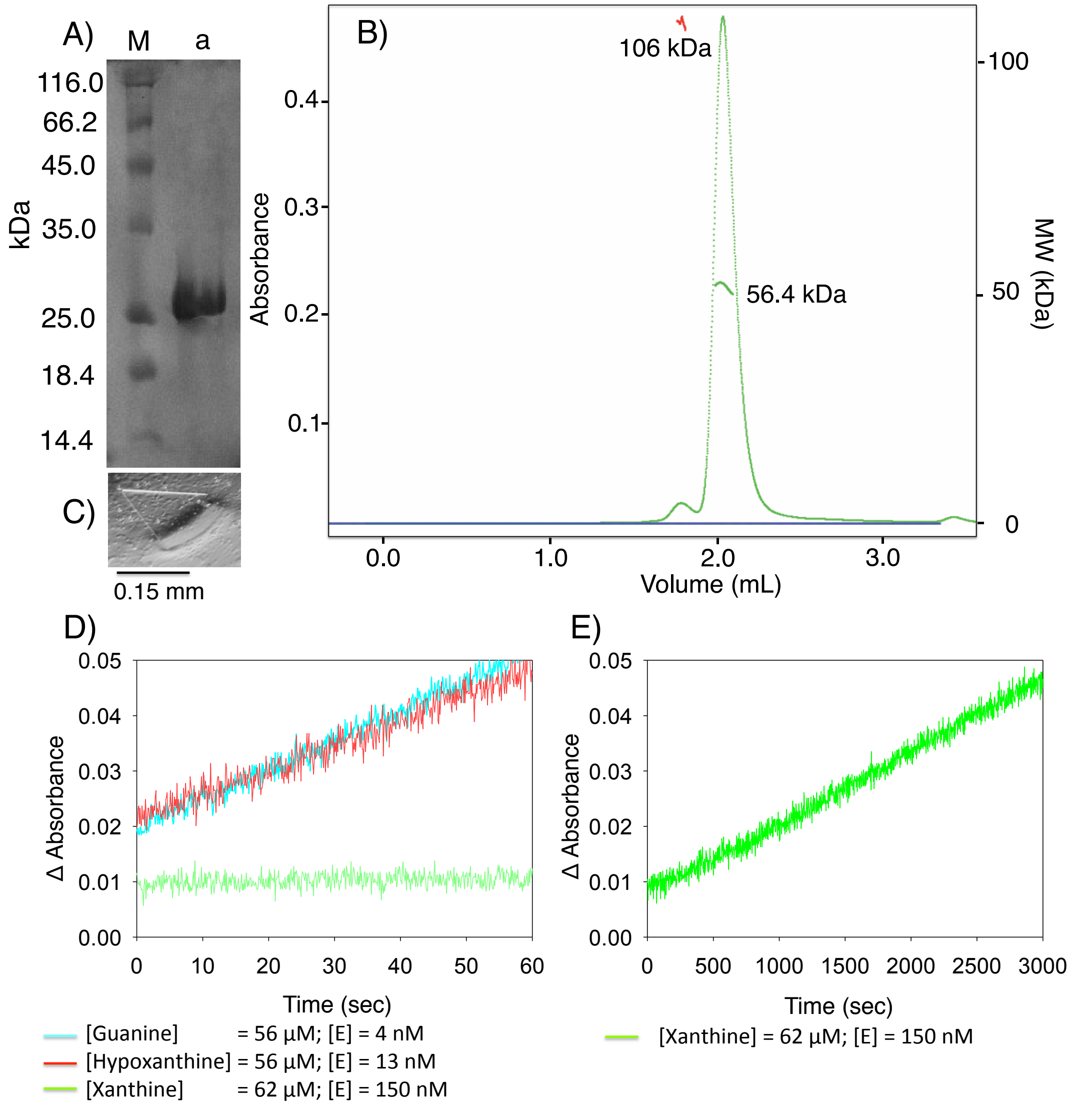

Supplementary Figure 1. Expression, purification, characterization and crystallization of *Tbr*HGPRT. A) 12% SDS-PAGE gel M: marker, a: purify *Tbr*HGPRT after dialysis. B) SEC-MALLS analysis for the green line represents the elution of protein by molecular weight. C) Crystal obtained in complex with **6**. D) and E) Spectrophotometric measurement of the rate of absorbance for the three naturally occurring purine base substrates with *Tbr*HGPRT. The Δε values for conversion of hypoxanthine to IMP, guanine to GMP and xanthine to XMP at 245, 257.5, 255 nm are 2439 M^{-1} cm^{-1}, 5817 M^{-1} cm^{-1} and 4685 M^{-1} cm^{-1}, respectively.

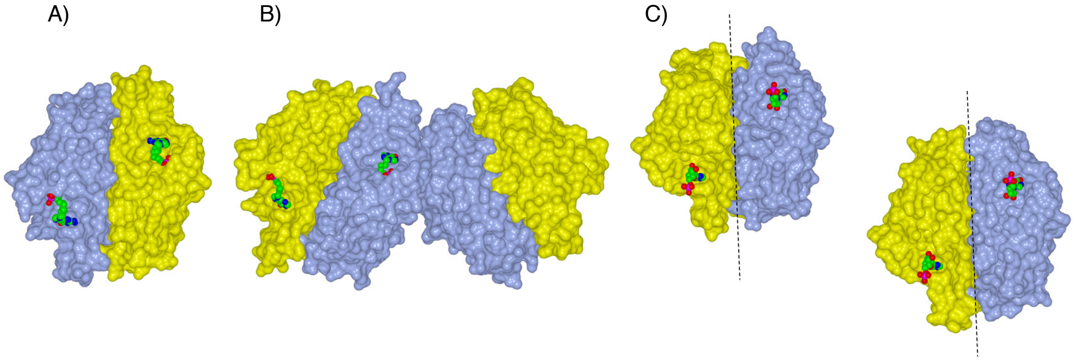

Supplementary Figure 2. The asymmetric unit for the three different space groups for *Tbr*HGPRT. A) The complex with **3**. The same packing is observed in the complex with **5**. B) The complex with **6** and C) The complex with IMP. The same packing is observed in the GMP complex.

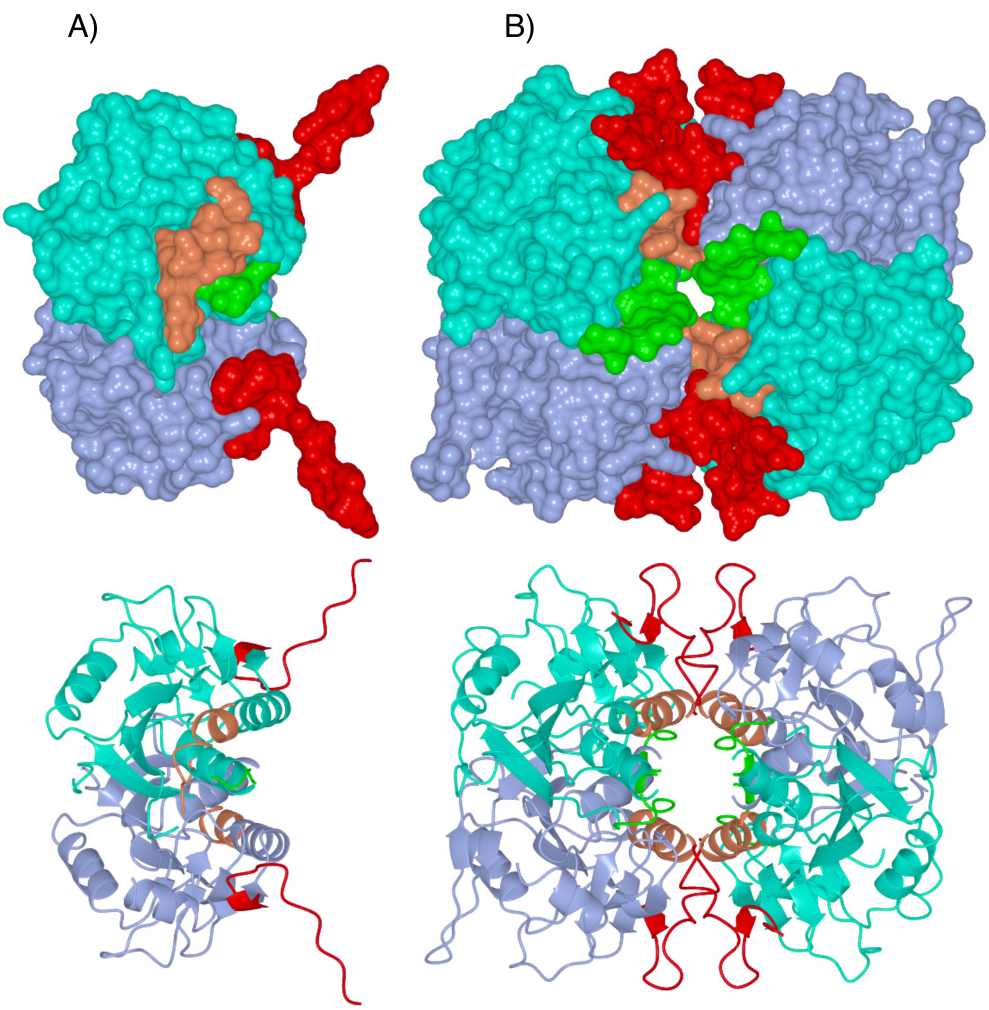

Dimer interface																	
**Human HGPRT**	P24		L67	K68	Y71	K72	D79	K82			R86	T96	V97	D98		E196	
***Tbr*HGPRT**		L43	L53	K54	F57	V58	R65	V76	E77	F78					Q176	R182	W195
**Human tetramer interface**																	
**Human HGPRT**	V8		E13	Y16	D19	L20	C22	G39	D43	R44	E46		R47	R50	L84	R86	N87
**Predicted tetramer interface based on human HGPRT**																	
***Tbr*HGPRT**	-		A4	Y7	A10	T11	V13	R30	D34	Y35	N37		C38	K41	F71	-	-

Supplementary Figure 3. Subunit associations in human HGPRT and *Tbr*HGPRT. (A) Connolly surface and ribbon representation of the *Tbr*HGPRT.GMP complex (B) Connolly surface and ribbon representation of the human HGPRT tetramer (PDB code: 3GEP). The cyan and blue represent the dimer pairs in each enzyme. The red, orange and green regions are where residues at the tetramer interface in human HGPRT. The corresponding residues in *Tbr*HGPRT are in identical colours and are listed in the table below.

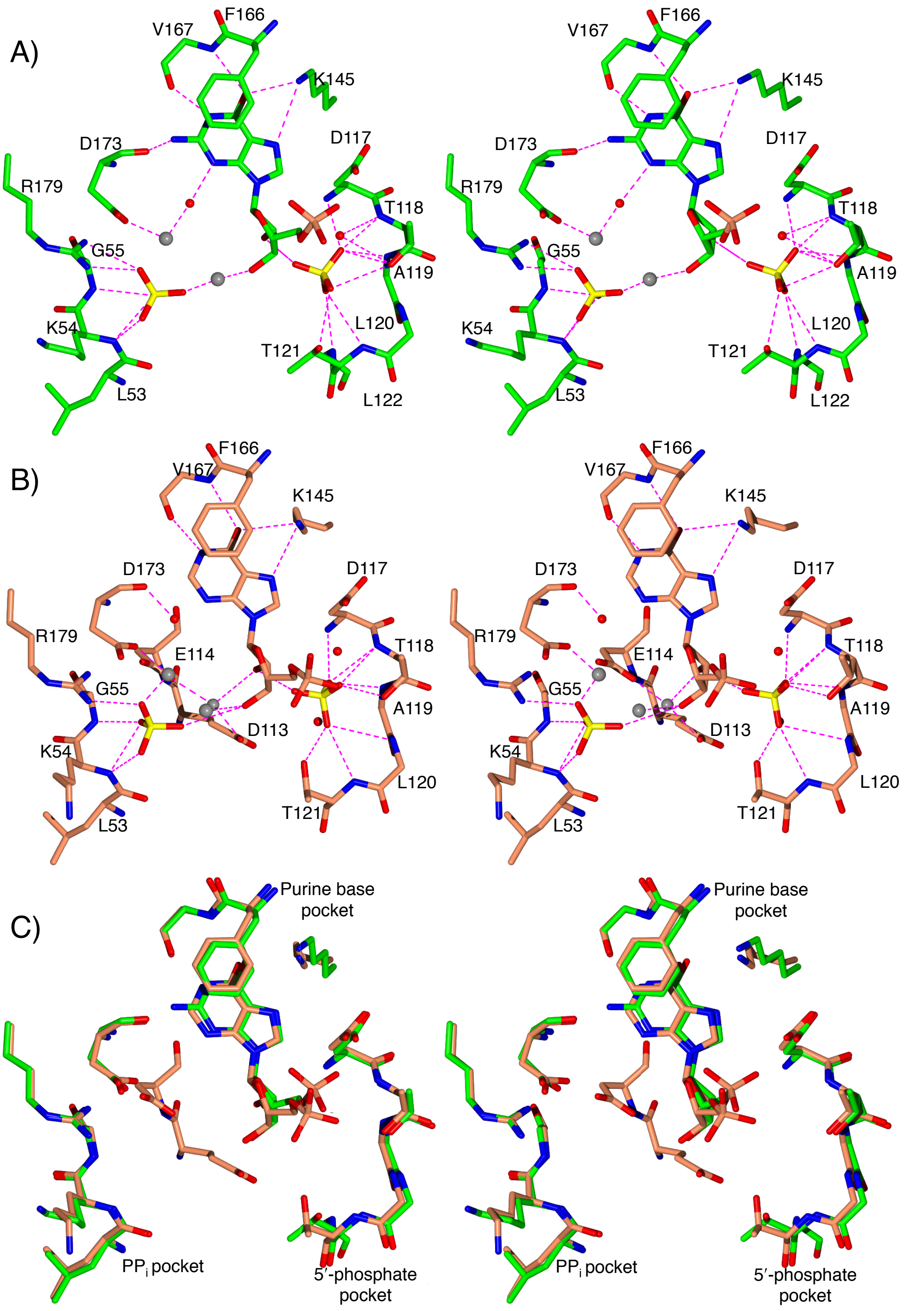

Supplementary Figure 4. Stereoview of the active site of *Tbr*HGPRT when in complex with the nucleotide products. A) Complex with GMP

(green carbon atoms), sulfate and Mg^{2+} ions (grey spheres). B) Complex with IMP (orange carbon atoms), sulfate and Mg^{2+} ions (grey spheres). C) Superimposition of the *Tbr*HGPRT.GMP and *Tbr*HGPRT.IMP complexes.

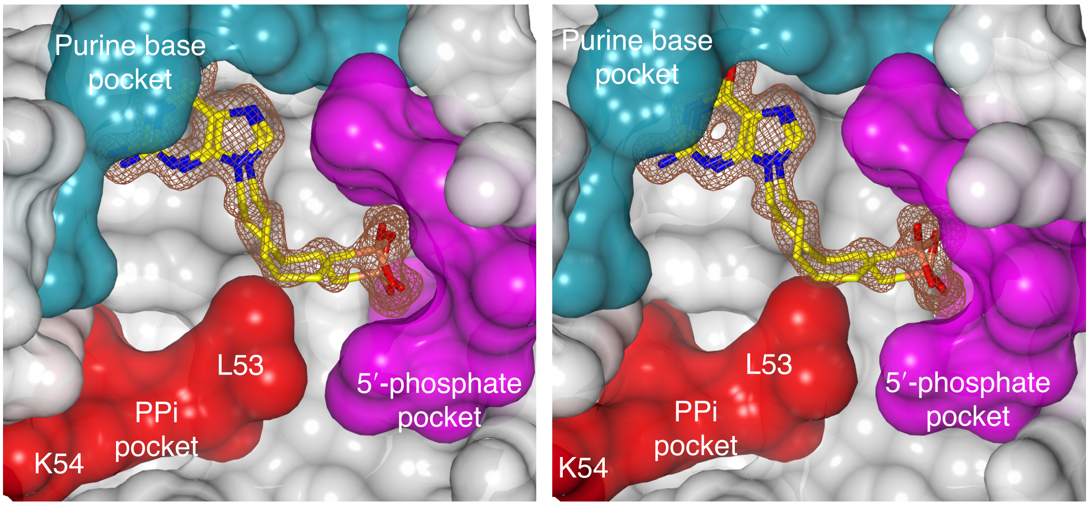

Supplementary Figure 5. Stereoview for the Connolly surface of the polypeptide for the *Tbr*HGPRT.3 complex. F_o-F_c "omit" electron density for **3**. This compound adopts two different conformations when bound to the enzyme.

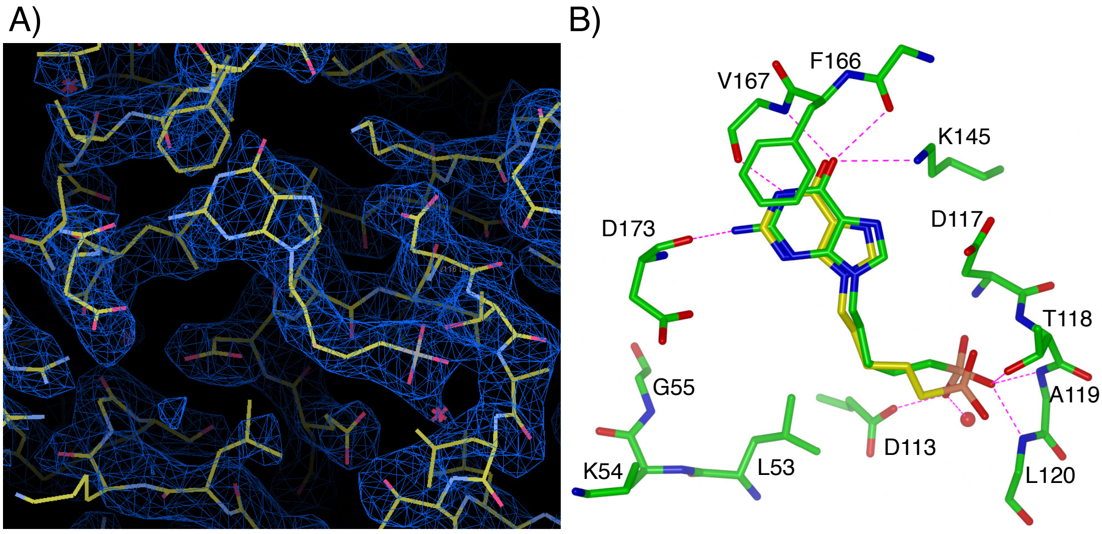

Supplementary Figure 6. The active site of the *Tbr*HGPRT.**2 complex.** A) $2F_o$-F_c electron density map B) Comparison of binding between **2** (green sticks) and **6** (yellow sticks).
